# Construction and application of an eye care plan for ICU patients receiving prone position ventilation: a quasi-experimental study

**DOI:** 10.3389/frhs.2026.1793496

**Published:** 2026-08-27

**Authors:** Ren Yan, Peng Fei, Xing Xiao-Hong, Li Yang-Yang, Yu He-Hua

**Affiliations:** 1 Nursing Department, Shanghai Changzheng Hospital, Shanghai, China; 2 Emergency and Critical Care Medicine Department of Shanghai Changzheng Hospital, The Second Affiliated Hospital of Naval Medical University, Shanghai, China

**Keywords:** prone position ventilation, intensive care unit, ocular complications, nursing care, evidence-based practice

## Abstract

**Objective:**

To develop an eye care plan for patients in the Intensive Care Unit (ICU) undergoing prone position ventilation (PPV), and to preliminarily evaluate its clinical application effect.

**Method:**

This study constructed an eye care plan for patients undergoing PPV in ICU based on evidence synthesis and expert consultation. Using convenience sampling method, this study established an intervention group (from August to November 2024) and a control group (from April to July 2024). Patients in the intervention and control groups received care based on the developed eye care plan and routine eye care, respectively, for one week. The incidence of ocular complications and changes in intraocular pressure (IOP) were compared between the two groups after the observation period.

**Results:**

A total of 58 patients were included, with 29 in each group. There were no significant differences in baseline characteristics between the two groups. The total incidence of ocular complications within one week decreased from 27.6% to 6.8%. The comparison of intraocular pressure within one week showed that the intraocular pressure of patients in the evidence-based practice group decreased after the 8 h, and the difference was statistically significant (*P* < 0.05).

**Conclusion:**

The implementation of an eye care plan for patients undergoing PPV in ICU can effectively reduce the incidence of ocular complications, alleviate IOP fluctuations, and improve the quality of life of patients eventually.

## 1 Introduction

Prone position ventilation (PPV) refers to a therapeutic modality enabling the promotion of lung expansion and improvement of oxygenation by changing the patient's position taking advantage of the gravity. PPV has been widely used in ICU in recent decades (1). However, patients may experience ocular compression during prolonged prone positioning, and the use of sedative drugs can impair ocular defense mechanisms, causing physiological changes such as increased intraocular pressure (IOP). Consequently, a series of complications may occur, including globular chemosis exposure keratitis, acute angle-closure glaucoma, and orbital syndrome, with an estimated incidence of 23% -60% (2, 3), which can seriously affect patients' the quality of life (4, 5). Current guidelines, both domestic and international, recommend routine care such as maintaining eye exposure, providing lubrication, and avoiding ocular compression (6, 7). However, targeted, practical, and standardized clinical protocols are poorly explored. Accordingly, various eye intervention measures have been proposed by foreign scholars, aiming at reducing direct IOP increase and alleviating eye dryness (8). However, it is still a challenge to apply the above measures in China due to different medical environments. Domestic scholars have realized the necessity of eye care for PPV patients, yet without well-developed standardized and systematic specific protocols so far (9, 10). Therefore, this study aimed to develop an eye care plan for patients undergoing PPV in ICU and to evaluate the preliminary validation. The findings are expected to provide potential practical guidance for clinical practice in this field, and to improving nursing quality and prognosis of PPV patients.

## 2 Method

### 2.1 Construction of eye care plan for patients receiving PPV in ICU

#### 2.1.1 Establishment of a research group of eye care

**Figure 1:**
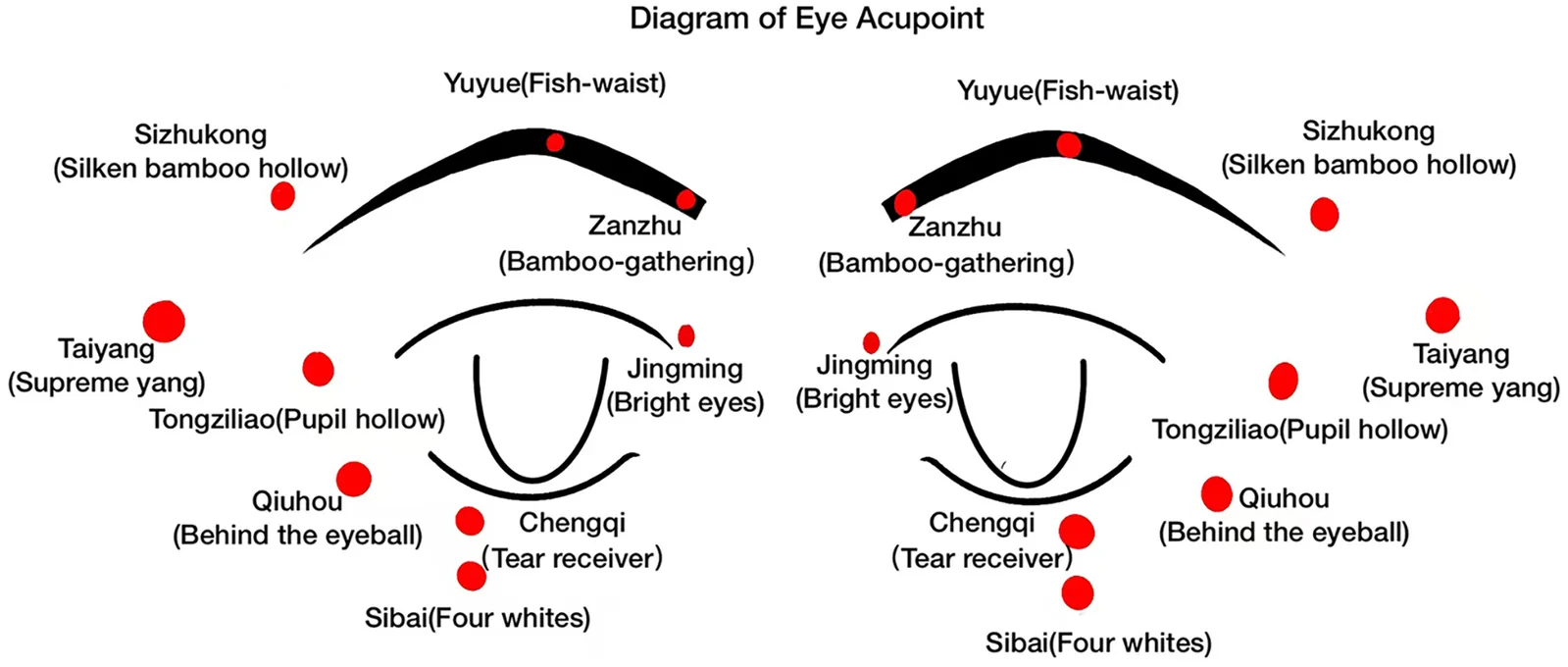
Eye acupoint massage.

**Figure 2:**
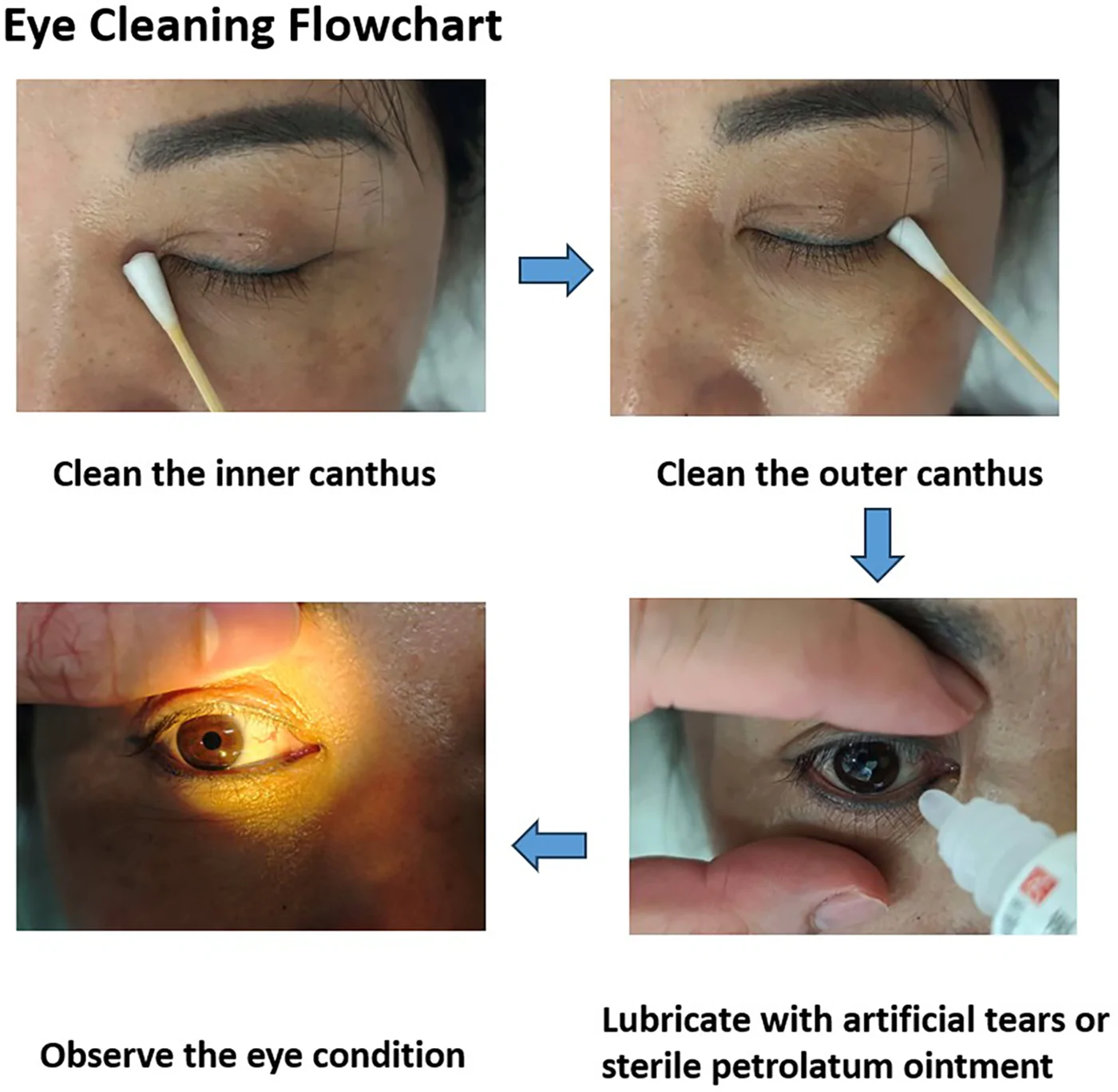
Eye cleaning.

Initially, this study established an ICU PPV patient eye care plan construction team (hereinafter referred to as the “Plan Construction Team”), consisting of 7 members totally, with one director of the Nursing Department (Figure 1) being responsible for research plans organization and pl anning (Figure 2), as well as project guidance; one chief head nurse for quality control during plan formulation and implementation; two head nurses for plan process development, personnel rescheduling, and plan implementation; and three master postgraduates in nursing for data collection, organization, and analysis.

#### 2.1.2 Summary of evidence on eye care

##### 2.1.2.1 Retrieval strategy

The specific questions of evidence summary were constructed using the PIPOST model (Table 1) (11). The PIPOST model is a structured framework commonly used in evidence-based practice to formulate clinical questions and guide evidence retrieval. It includes six components.

**Table 1:** Proposed PIPOST model.

PIPOST model	Description
P (Population)	Patients who were admitted to ICU and underwent PPV.
I (Intervention)	Specific nurse-led intervention for preventing or treating ocular complications
P (Professional)	Clinical nursing staff
O (Outcome)	Incidence of ocular complications
S (Setting)	Intensive Care Unit (ICU)
T (Types of evidence)	Clinical decision-making, evidence summaries, guidelines, and systematic reviews

Based on the “6S” evidence model, evidence retrieval was conducted in a hierarchical manner (12). The 6S model is an evidence-based resource pyramid proposed by Haynes, which organizes evidence sources into six levels, including systems, summaries, synopses of syntheses, syntheses, synopses of studies, and original studies. This model facilitates efficient and systematic retrieval of the best available evidence by prioritizing high-level synthesized evidence before primary studies. Specifically, the following databases and websites were searched as follows: (1) evidence-based guidelines database, including the National Guideline Clearinghouse in the United States, the Guidelines International Network, the National Institute for Health and Clinical Excellence Guidelines in the United Kingdom, the Scottish Intercollegiate Guidelines Network, and the Registered Nurses Association of Ontario Guideline in Canada. (2) Authoritative professional association websites, covering the American Association of Critical-Care Nurses, European Society of Intensive Care Medicine, Intensive Care Physician Branch of the Chinese Medical Doctor Association, American Academy of Ophthalmology, Royal College of Ophthalmology, and Faculty of Intensive Care Medicine in the UK. (3) Pre-evaluation evidence databases, such as Cochrane Library, Joanna Briggs Institute (JBI) Evidence Summary Library, BMJ Best Practice, and UpToDate database. (4) Comprehensive biomedical literature databases, involving PubMed, Embase, CINAHL (EBSCO), Web of Science, China National Knowledge Infrastructure, Wanfang Database, VIP Database, China Biology Medicine Database, and Medlive Database. In addition, the Metz Medical website, Baidu Scholar, and Google Scholar were also searched to expand the literature included in our study as much as possible. The search terms consisted of theme-words and keywords jointly. Both English and Chinese databases were searched using equivalent search terms related to prone position ventilation, intensive care unit, ocular complications, nursing care, and evidence synthesis. The retrieval time for all databases and websites was from the establishment of the database to November 2023. Taking PubMed as an example the detailed search strategy is presented in Supplementary File S1.

##### 2.1.2.2 Inclusion and exclusion criteria for eligible studies

Inclusion criteria: (1) Study subjects: adult patients receiving PPV in ICU; (2) Intervention measures: specific nursing measures (e.g., eye assessment, eye care, complication management) implemented by ICU nursing staff to prevent and treat ocular complications related to PPV; (3) Outcome indicators: incidence and severity of ocular complications patients receiving PPV within ICU; (4) Research types: evidence-based guidelines, expert consensus, systematic review/meta-analysis, randomized controlled trials (RCTs), and evidences of other different levels; and (5) Language: literature in Chinese or English. Exclusion criteria: (1) duplicates; (2) literature with no full-text such as conference abstracts and paper abstracts, and literature without access to the full-text; (3) Research on special populations such as newborns and children; and (4) Literature with lower levels of evidences such as case reports, expert advice, and forum discussions.

##### 2.1.2.3 Literature screening, quality evaluation, and evidence level

The quality of the included literature was evaluated using different quality assessment tools. Specifically, the AGREE II (Appraisal of Guidelines for Research&Evaluation II) tool was adopted for quality evaluation of evidence-based guidelines (13). The expert consensus evaluation developed by JBI was used to realize quality evaluation of expert consensus (14). The AMSTAR 2 tool was selected for system evaluation/meta-analysis quality evaluation (15). Cochrane bias assessment tool was used for quality evaluation of RCTs (16).

#### 2.1.3 Expert consultation

##### 2.1.3.1 Expert selection

Inclusion criteria for experts: (1) Bachelor's degree or above; (2) professional title of medium-grade or above; (3) Over 10 years of experience in nursing or medicine; and (4) relevant experience in PPV.

##### 2.1.3.2 Expert meetings

Relevant materials were distributed to the included experts after obtaining their consent prior to the meeting, and the time and place were subsequently confirmed with them. After the selection of the expert group leader, the researchers explained the purpose, content, and relevant materials on the day of the meeting. Following researchers' presentation, the researchers answered the experts' questions and recorded their suggestions or opinions one by one. Afterwards, a detailed discussion was organized by the expert group leader to evaluate the scientific, applicability, and feasibility of the initial draft of the plan, and corresponding revision suggestions were provided. After the meeting, the researchers summarized the results to the experts, and the meeting ended when no objections were raised.

##### 2.1.3.3 Development of the final eye care plan

Based on expert consultation, the preliminary eye care plan was revised and refined in terms of assessment criteria, nursing procedures, infection control requirements, and implementation frequency. To be specific, regarding the eyelid closure ability in the eye assessment, experts pointed out that “it can be evaluated based on the eyelid closure ability assessment form recommended in the Australian Guidelines for Eye Care for Critically Ill Adults”. For the development and implementation of nursing standards for the prevention and management of ocular complications in patients undergoing PPV in ICU, experts suggested that the original statement was vague and should be changed to “develop and implement standardized nursing procedures and emergency plans for ocular complications in patients undergoing PPV in ICU”. Meanwhile, concerning eye acupoint massage, experts mentioned that hand hygiene should be performed before procedure due to infection control requirements. In addition, experts pointed out that the description of the implementation frequency and location of nursing evaluation and eye cleaning, such as “at fixed time” and “at fixed place” were vague and not conducive for clinical implementation. Considering that eye care was scheduled every 4 h in other nursing measures, this was revised to “They should be integrated during the practice process, and hence eye evaluation, eye cleaning, and care are all implemented every 4 h”. The finalized eye care plan is presented in Supplementary File Table S1.

### 2.2 Application of eye care plan for patients receiving PPV in ICU

#### 2.2.1 Study design and participant

**Figure 3:**
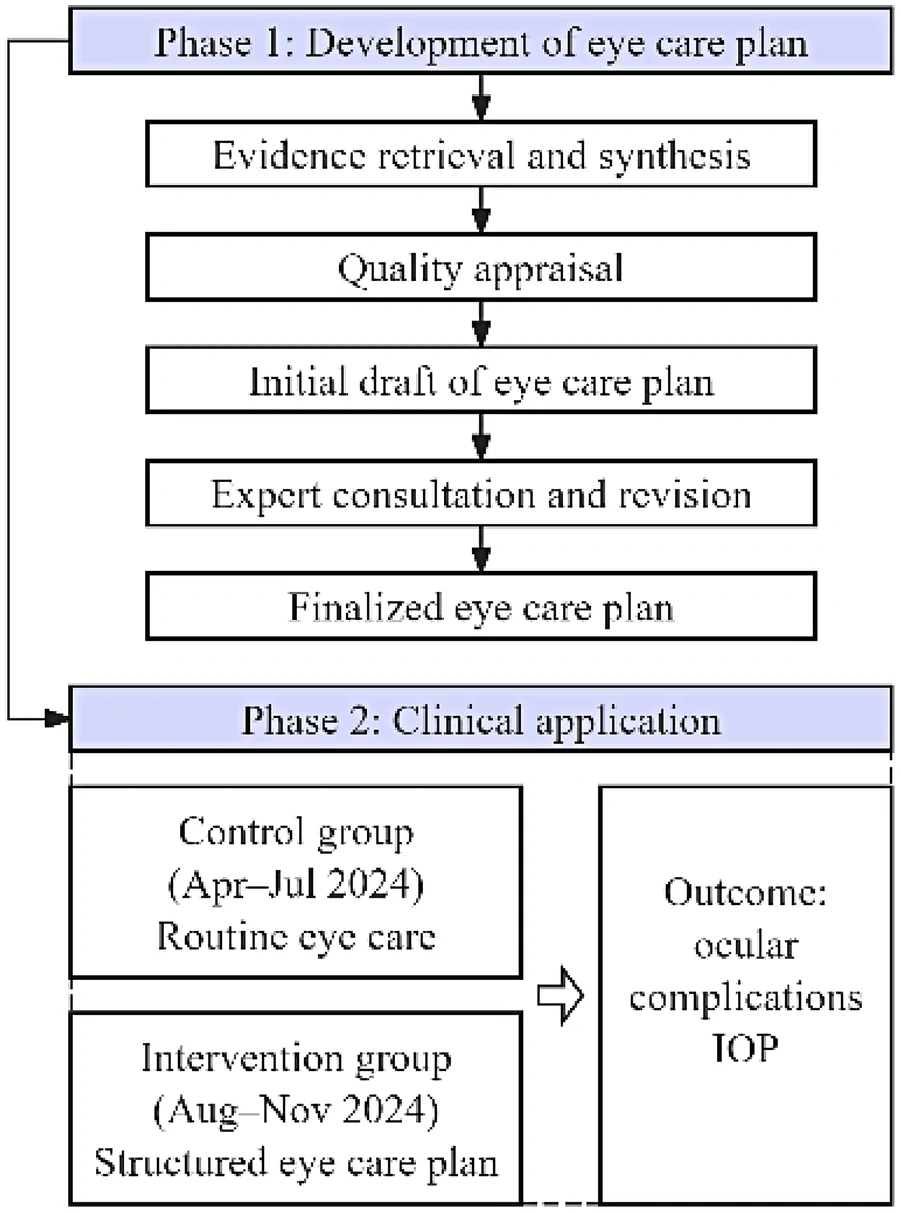
The study process.

We conducted a quasi-experimental design to evaluate the effect of eye care plan. Convenient sampling approach was used to enrol the participants from ICU, who were receiving PPV. The control group enrolled from April to July 2024 and the intervention group from August to November 2024. The study process is presented in Figure 3.

Inclusion criteria: (1) patients who could undergo PPV and receive medical advice based on the doctor's judgment; (2) patients who aged >18 years old; (3) patients who voluntarily or with written informed consent provided by their family members; and (4) patients with calm consciousness [Richmond Agitation-Sedation Scale (RASS) score of −3 to −4 points] (24). Exclusion criteria: (1) patients with severe hemodynamic instability; (2) patients with acute hemorrhagic diseases; (3) patients with cervical and spinal injuries; (4) patients with unstable fractures; (5) pregnant women; (6) patients who needed to undergo surgery with restricted positions in the near future; (7) patients who could not tolerate prone position; (8) patients undergoing surgeries for facial and eye injuries; (9) patients who did not participate in the study voluntarily or with their family members unwilling to participate; and (10) patients with eye diseases or having undergone surgery. Criteria for dropout: (1) patients unable to continue the study due to serious complications; (2) patients who died or were transferred to other departments or hospitals during the research period; and (3) patients unable to continue to participate in this study due to personal reasons.

The following parameters were used for sample size calculation: *α*=0.05 (two-sided test) and *1-β*=0.8 based on the two-sided binomial test, as well as *Z_1_-α*＝1.96, *Z_1_-β*＝0.84, *p_1_* = 6.0%, and *P_2_* = 26.0% from literature review. According to the calculation, 29 cases were respectively required for both the intervention group and the control group considering a 10% dropout rate, with 58 patients needed totally. Finally, 58 patients were included in this study, with 29 in each group. The study was approved by the hospital's ethics committee, and all participants provided informed consent and participated voluntarily.

#### 2.2.2 Personnel training

Medical staffs were trained to enhance their theoretical knowledge and practical skills in eye care for PPV patients through various forms and multi-level training strategies. Theoretical training (theme lectures, case sharing, online learning) included basic knowledge of PPV in ICU, key points of eye assessment, identification and treatment of common ocular complications. Centralized training was conducted once every two weeks for 2 h each time. Meanwhile, practical training covered eye care Standard Operating Procedure (SOP), IOP measurement, acupoint massage, emergency response procedures for complications. Via scenario simulation and on-site demonstration, skills training sessions were held once a month for 3 h each time. Theoretical exams, skill assessments were employed for evaluation after training completion, and only those who passed were qualified for clinical practice. The effectiveness of training was monitored and evaluated monthly by the quality control team. Training content was dynamically adjusted based on feedback collected through on-site questioning, nursing rounds, and medical discussions.

#### 2.2.3 Intervention methods

The control group adopted routine eye care, including eye condition assessment, eye cleaning and basic positioning adjustments every 4 h. The intervention group received care based on the developed and structured eye care plan. The simple assessment mainly included signs of ocular complications such as eyelid edema, purulent discharge in the eyes, eye congestion, etc. For cleaning, the eyes were wipe with a cotton ball dipped in sterile physiological saline from the corner to the end of the eye. Moreover, patients in prone position should be turned left or right every 4 h, with attention paid to exposing the eyes to avoid eye compression. The comparison of the eye care was shown in Table 2.

**Table 2:** Comparison of eye care between groups.

Feature	Control group	Intervention group
Organizational Management	General ICU routine nursing care	(1) Standardized procedures (2) Establish MDT (16) Quality control (17) Special circumstances (18) Shift management
Before PPV	(1) Initial Evaluation: simple evaluation process including ocular complications (2) Eye cleaning: routine eyes wipe	(3) Initial Evaluation: comprehensive evaluation process including ocular complications, eyelid closure ability, influencing factors of iatrogenic (4) Eye cleaning: eyes wipe with artificial tears or sterile Vaseline eye ointment can be applied to maintain lubrication (5) Eye acupoint massage (6) Periocular protective pad
During PPV	(3) Position: Left/right turns every 4 h to expose eyes and avoid compression (4) Eye care: routine eyes wipe (5) Disease observation: routine ocular complication	(7) Position: 30° head elevation; use of gel headrests and depressed foam pads to keep eyes suspended; eye masks if necessary (8) Closed sputum suction (9) Oral and nasal secretion cleaning (10) Eye care: eyes wipe with artificial tears or sterile Vaseline eye ointment (11) Disease observation: comprehensive symptom observation and coordinate with MDT immediately if complication occur
After PPV	(6) Eye assessment (7) Complication management: rountine (8) Eye cleaning: routine eyes wipe	(12) Eye assessment (13) Complication management: comprehensive with MDT (14) Eye medication (15) Eye cleaning: eyes wipe with artificial tears or sterile Vaseline eye ointment and eye acupoint massage

### 2.3 Evaluation methods

#### 2.3.1 Incidence of ocular complications in patients undergoing PPV in ICU

In our study, the ocular complications were identified to be chemosis, conjunctival congestion, corneal injury, exposure keratitis, acute closed glaucoma, and orbital syndrome based on existing studies (5, 9, 10). All complications were diagnosed by one physician in ICU and one ophthalmologist, and recorded by the nurse. In general, ocular complications would occur within one week after admission (17, 18), therefore, the observation period in this study was set as one week. The incidence of complications was calculated as follows: incidence (%) = (number of patients with complications during the observation period ÷ total number of patients included in the observation) × 100%.

#### 2.3.2 IOP of patients receiving PPV in ICU

In our study, IOP of patients receiving PPV in ICU was measured by using an independently designed patient IOP statistical table. It has been recognized that there was a positive correlation between patient IOP and prone position time (19); meanwhile, a prone position time of 12–16 h is recommended by the guidelines (20). Based on this rationale, IOP measurements were scheduled at predefined time points: before PPV, at 4 h, 8 h and 16 h of PPV, and at the end of PPV. The left and right eyes were measured separately using a German Icare ic100 tonometer. To ensure data accuracy and clinical safety, the nursing staff followed a standardized “Load-Align-Measure” protocol: (1) loading: the probe base cover was removed, and a new disposable probe was inserted into the probe base to prevent cross-infection; (2) alignment: the probe was maintained in a horizontal position and aligned vertically with the center of the patient's cornea. The tip of the probe was kept at a distance of 4–8 mm from the cornea. A built-in position indicator light turned green when the probe was correctly positioned, signaling that measurement could proceed; (3) measurement: the device was operated in “Single Model”, where a short press of the button obtained a single measurement result; (4) auditory feedback: the device provided real-time feedback through beeps. One short beep indicated a successful measurement, while two short beeps indicated an error, necessitating a re-measurement. To further ensure reliability, nurses gently separated the eyelids during the procedure to avoid applying external pressure to the eyeball. Each eye was measured three times during every assessment turn, and the average value of these three readings was recorded as the final result. The device was used according to the manufacturer's instructions, and disposable probes were used for each measurement to prevent cross-infection. For patients presenting with contraindications such as active eye infections, the measurement was paused and documented in detail.

#### 2.3.3 Data collection methods

Data collection was conducted using a self-designed questionnaire, including general information, eye conditions (IOP, complications, etc.), and nurses' operation. General information (e.g., gender, age, diagnosis, Acute Physiology and Chronic Health Evaluation II score, PPV time, etc.) was filled in by the responsible nurse at the time of patient enrollment. The patient's eye condition was evaluated and recorded daily by the responsible nurse, covering abnormal manifestations of the cornea, conjunctiva, eyelids, eyelids, and ocular secretions. For patients who developed ocular complications, an ophthalmologist was consulted in a timely manner to confirm the diagnosis, and the type, severity, and outcome of the complications were recorded in detail.

#### 2.3.4 Statistical analyses

SPSS 26.0 statistical software was used for data organization and analysis. Measurement data with a non-normal distribution was represented by median (upper and lower quartiles), and intergroup comparisons were performed Mann–Whitney U test. The generalized estimation equation model was used to comparison at a single time point were also conducted using the Mann–Whitney U test. Meanwhile, the Friedman test was used for intra-group comparison at multiple time points in a single group, and the Bonferroni test for pairwise comparison afterwards. Counting data was expressed in frequency (percentage), and corresponding inter-group comparison was performed using chi-square test, corrected chi-square test, or Fisher's exact test. Intra-group comparison utilized paired chi-square test. *P* < 0.05 indicated statistical significance of the difference.

## 3 Results

### 3.1 Results of evidence summary

A total of 8 studies were finally included after literature screening and quality appraisal, including 4 guidelines (7, 21–23), 1 expert consensus (24), 1 systematic review (25), 1 evidence summary (26), and 1 randomized controlled trial (27).

Among the included guidelines, 3 studies (7, 21, 22) were recommended as Grade A and 1 study (23) as Grade B according to the AGREE II evaluation criteria. The expert consensus study showed high methodological quality based on the JBI appraisal tool. The systematic review demonstrated acceptable quality according to the AMSTAR 2 assessment, while the randomized controlled trial showed generally low risk of bias despite unclear allocation concealment and blinding procedures.

Through evidence extraction and synthesis, 23 pieces of evidence were identified and summarized into five domains, including organizational management, eye assessment and cleaning before PPV, nursing interventions during PPV, management after PPV, and personnel training and education. Based on the summarized evidence, a preliminary eye care plan for patients undergoing PPV in ICU was developed.

### 3.2 Results of expert consultation

A total of 7 experts participated in the expert consultation, including one ICU physician, one ophthalmologist, one ophthalmic nurse, one traditional Chinese medicine physician, two ICU nurses, and one ICU nursing manager. Among them, 4 experts hold the title of associate professor or above, and five experts have master's or doctoral degree. The expert authority coefficient (Cr) was calculated to be within the range of 0.775–1.000, with the group authority coefficient of 0.868.

### 3.3 Comparison of general information between the two groups

A total of 58 patients (43 males and 15 females) were included in this study and randomly assigned to the control and intervention groups, with 29 cases in each group. As shown in Table 3, there was no statistically significant difference between groups (all *P* > 0.05).

**Table 3:** Comparison of general information between the two groups of patients (*n* = 58).

Indicator	Control group (*n* = 29)	Intervention group (*n* = 29)	*χ*^2^/*z* Value	*P-*Value
Gender (case)			0.090^b^	0.764
Male	22	21		
Female	7	8		
Age [Years, *M(Q_1_,Q_3_)*]	70.00 (64.50, 75.00)	70.00 (63.50, 72.00)	−0.335^a^	0.738
Diploma (case)			2.523^b^	0.283
Elementary school and below	13	17		
Middle school	9	4		
College and undergraduate	7	8		
Marriage status (case)			0.069^b^	0.792
Married	13	14		
Widowed	16	15		
BMI [kg/m^2^, *M(Q_1_,Q_3_)*]	20.00 (19.00, 21.50)	21.00 (19.50, 22.00)	−1.032^a^	0.302
APACHEII Score [point, *M(Q_1_,Q_3_)*]	22.00 (20.00, 25.00)	24.00 (22.00, 25.00)	−1.294^a^	0.196
Total number of PPV [time, *M(Q_1_,Q_3_)*]	2.00 (1.00, 2.00)	1.00 (1.00, 2.00)	−1.228^a^	0.220
Total time of PPV [Hour, *M(Q_1_,Q_3_)*]	18.00 (12.00, 20.00)	15.00 (12.00, 20.00)	−0.585^a^	0.559
Division time of PPV [hour, *M(Q_1_,Q_3_)*]	10.00 (10.00, 12.00)	10.00 (10.00, 12.00)	−1.105^a^	0.269
RASS Score [point, *M(Q_1_,Q_3_)*]	−3.50 (−4.00, −3.00)	−3.50 (−4.00, −3.00)	−0.198^a^	0.843

^a^ represents Mann–Whitney *U*-test, ^b^ represents chi square test, and ^c^ represents Fisher's exact test.

### 3.4 Comparison of the incidence of ocular complications

As presented in Table 4, there was no statistically significant difference in the incidence of chemosis, exposure keratitis, and corneal injury between groups within one week (*P* > 0.05). However, the total incidence of ocular complications was lower in the intervention group than that in the control group (*P* < 0.05). Therefore, it could be speculated that the application of this care plan can contribute to the reduction of the total incidence of ocular complications.

**Table 4:** Comparison of total incidence of complications between the two groups of patients [example (percentage, %)].

Indicators	Control group (*n* = 29)	Intervention group (*n* = 29)	χ2 Value	*P*-Value
Chemosis	4 (13.8)	1 (3.4)	-b	0.352
Exposure keratitis	2 (6.9)	1 (3.4)	-b	1.000
corneal injury	2 (6.9)	0 (0)	-b	0.491
Total incidence rate	8 (27.6)	2 (6.8)	4.350a	0.037

a represents chi square test, b represents Fisher's exact test.

### 3.5 Comparison of IOP

According to the comparison of IOP between groups within one week (Tables 5 and 6), both left and right IOP in the intervention group decreased after 8 h and were lower than those in the control group, with statistically significant difference (*P* < 0.05). Consequently, the application of this care plan can control IOP fluctuation in patients within a certain range, thereby reducing the occurrence and development of ocular complications.

**Table 5:** Comparison of left IOP changes between two groups of patients [mmHg, *M(Q1,Q3)*].

Groups	Cases	Before	4 h	8 h	At the end	*χ*^2^ Value	*P*-Value
Control group	29	18.00 (16.50,22.50)	23.00 (20.00,25.00)^a^	29.00 (25.00,33.50)^a^	25.00 (22.75,28.75)^a^	73.133	<0.001
Intervention group	29	20.00 (17.00,22.50)	22.00 (20.25,24.50)^a^	23.00 (22.00,31.00)^a^	22.50 (20.00,25.50)^a^	49.734	<0.001
*z* Value		−0.117	−0.827	−2.396	−2.136		
*P*-Value		0.907	0.408	0.017	0.033		

Compared to the value before PPV, ^a^ *P* *<* *0.05*.

**Table 6:** Comparison of changes in right IOP between the two groups of patients [mmHg, *M(Q1,Q3)*].

Groups	Cases	Before	4 h	8 h	At the end	*χ*^2^ Value	*P*-Value
Control group	29	17.00 (16.00,21.00)	23.00 (21.00,25.00)^a^	30.00 (26.50,32.65)^a^	25.00 (22.50,28.00)^a^	69.253	<0.001
Intervention group	29	19.00 (15.00,21.00)	22.00 (21.00,23.75)^a^	25.50 (21.50,31.50)^a^	22.50 (21.00,24.00)^a^	52.371	<0.001
*P*-Value		−0.031	−1.346	−2.505	−2.416		
Control group		0.975	0.178	0.012	0.016		

Compared to the value before PPV, ^a^ *P* *<* *0.05*.

## 4 Discussion

### 4.1 Strong scientificity and operability of the constructed eye care plan for patients receiving PPV in ICU

This study identified evidence-based issues through literature review in the early stage. Eventually, 23 pieces of evidence covering organizational management, risk identification, preventive measures, intervention treatment, as well as training and education were developed by integrating evidences from relevant guidelines, systematic reviews, expert consensus, and RCTs at home and abroad. Subsequently, each item of the initial care plan was revised through expert group meeting. During optimization, the specific clinical context in China was fully considered, and valuable experience from clinical nursing and medical experts was incorporated, ultimately forming the eye care plan for patients receiving PPV in ICU. The plan clearly formulated evidence-based procedures for eye care management, providing standardized operational guidance for nurses. Moreover, it emphasized targeted training and education to strengthen risk awareness and enhance the initiative of standardized operations. In addition, the implementation status was included as an indication for the assessment to strengthen process management and continuous improvement. Therefore, this plan exhibits strong scientific and operability.

### 4.2 Effect of implementing the eye care plan on reducing the incidence of ocular complications for patients receiving PPV in ICU

Patients receiving PPV in ICU often have eye problems such as chemosis and exposure keratitis, with an incidence rate of 23%–60%. Severe cases may develop corneal ulcers, perforation, or even permanent vision loss leading to poor prognostic outcomes. Standardized and systematic nursing interventions have been proven to be an effective strategy to reduce the risk of complications (20). In our study, after implementing eye care plan, the overall incidence of ocular complications decreased from 27.6% to 6.8%, including chemosis from 13.8% to 3.4%, exposure keratitis from 6.9% to 3.4%, and corneal injury from 6.9% to 0%. Therefore, this plan can effectively prevent the occurrence of ocular complications in patients undergoing PPV in ICU. As for the potential reasons, it can be explained as follows: (1)this plan improved identification of risk factors by developing standardized eye assessment processes based on existing literature; (2) routine nursing procedures were revised according to evidence-based measures to reduce omissions and errors; (3) quality management was strengthened through regular complication screening, and objective evaluation of nursing effectiveness; and (4) interdisciplinary cooperation improved consultation and treatment processes, enabling timely control of disease progression. Similarly, Li Chunlin (28). have implemented an evidence-based eye care program for neurosurgical patients and reported a reduction in keratitis incidence from 38.2% to 17.9%. Therefore, findings in this study may provide new evidence to support the positive role of evidence-based nursing in preventing ocular complications in ICU patients.

### 4.3 Effect of implementing the eye care plan on alleviating IOP fluctuations for patients receiving PPV in ICU

Elevated IOP is a common physiological change in patients undergoing PPV. The maintenance of a prone position may result in ocular compression, impaired venous return, reduced aqueous drainage, and increased IOP by 18.9% eventually (19). Continuous increase in IOP can lead to ischemia and hypoxia in tissues such as the retina and optic nerve, exacerbating damage to the ocular surface (29, 30). These findings highlight the importance and necessity of effective control of patients' IOP for preventing secondary ocular injury. Clinical observations in this study demonstrated that IOP levels after 8 h in the prone position were significantly lower following the intervention, indicating that the implementation of this eye care plan can help alleviate the abnormal increase in IOP caused by PPV. It is speculated that: (1) elevating the head of the bed by 30° may improve venous return; (2) eye acupoint massage can promote aqueous drainage; and (3) ensuring eye exposure can avoid local compression (31). Through a comprehensive retrieval of relevant literature, this study constructed an eye care plan systematically, which can provide more comprehensive and scientific practical guidance for controlling IOP in patients undergoing PPV in ICU.

### 4.4 Limitations

The findings of this study should be interpreted in light of several limitations. First, the research was conducted at only one hospital, which may limit the generalizability of the results to other medical settings. Second, the study employed a quasi-experimental design and convenience sampling approach with a small sample, which may introduce potential selection bias compared to randomized controlled trials Third, while the overall protocol was traced by a quality control team, we did not perform a detailed multivariate analysis to isolate the independent impact of each of the 18 specific care points. Future research should focus on large-scale, multi-center studies to further validate, modify, and refine the eye care plan for broader clinical application.

## 5 Conclusion

This study successfully developed a standardized and systematic eye care plan for ICU patients receiving PPV by integrating evidence-based literature with expert consultation. The clinical application of this plan demonstrated a significant reduction in the total incidence of ocular complications and effectively alleviated abnormal fluctuations in IOP. By providing clear, practical guidance for ICU nurses, the care plan could facilitate nursing quality and improves patient prognosis.

## Data Availability

The original contributions presented in the study are included in the article/Supplementary Material, further inquiries can be directed to the corresponding author.

## Supplementary material

The Supplementary Materialfor this article can be found online at: https://www.frontiersin.org/articles/10.3389/frhs.2026.1793496/full#supplementary-material
