# Supplementary material for: Construction and application of an eye care plan for ICU patients receiving prone position ventilation: a quasi-experimental study
Source: Front Health Serv. 2026 Aug 27;6:1793496. doi: 10.3389/frhs.2026.1793496 (PMC13558220; doi:10.3389/frhs.2026.1793496)
Supplement: Supplementary file 1 [file Supplementaryfile1.docx]

An example of the the search strategy in PubMed:

The main search terms were as follows(“Prone Position” [Mesh] OR prone position* [Title/Abstract] OR PPV [Title/Abstract]) AND (“Intensive Care Units” [Mesh] OR “Critical Care” [Mesh] OR “Critical Illness” [Mesh] OR ICU [Title/Abstract] OR intensive care [Title/Abstract] OR critical care [Title/Abstract] OR critically ill [Title/Abstract]) AND (“Eye Diseases” [Mesh] OR eye [Title/Abstract] OR ocular [Title/Abstract] OR cornea* [Title/Abstract] OR conjunctiv* [Title/Abstract] OR eyelid [Title/Abstract]) AND (complication* [Title/Abstract] OR keratopathy [Title/Abstract] OR injur* [Title/Abstract] OR edem* [Title/Abstract]) AND (nursing [Title/Abstract] OR nurs* [Title/Abstract]) AND (guideline [Publication Type] OR consensus [Title/Abstract] OR practice guideline [Publication Type] OR systematic [Title/Abstract] OR meta-analy* [Title/Abstract])

Table 1 Construction of Eye Care Plan for Patients Receiving PPV in ICU

| Categories | Content of the plan |
| --- | --- |
| Organizational management | 1. Standardized procedures: Develop protocols and emergency plans for managing ocular complications. |
|  | 2. Establish multidisciplinary team (MDT): Form an interdisciplinary team (physicians, nurses, ophthalmologists) with defined roles for daily rounds, care implementation, and complication diagnosis. To be specific, ICU physicians are responsible for daily clinical assessment, particularly medication management; ICU nurses deliver eye care and monitor ocular status; ophthalmologists diagnose and manage ocular complications; and rehabilitation physicians support functional recovery in stable patients. |
| Before PPV | 3. Initial Evaluation: Assess patients upon ICU admission and at least twice daily thereafter. Increase frequency if prone duration exceeds 12 hours or during deep sedation. Evaluation includes IOP, eyelid closure ability, influencing factors of iatrogenic ocular complications, and ocular complications. The normal range of IOP is 10-21mmHg according to the International Association of Ophthalmologists standard. Eyelid closure is graded as complete, mild (<1 mm), moderate (1–2 mm), or severe (>2 mm); The influencing factors of iatrogenic ocular complications include patient-related factors (e.g., age >60 years, prolonged bed rest, diabetes, Parkinson’s disease, facial paralysis), medication use (e.g., sedatives, muscle relaxants, anticholinergics), and organ dysfunction (e.g., impaired eyelid closure, reduced tear secretion); The diagnostic criteria and grading references for iatrogenic ocular complications are as follows: Corneal exposure area: mild (<1/3), moderate (1/3~1/2), and severe (>1/2); Chemosis edema range: mild (<1/4), moderate (1/4-1/2), and severe (>1/2); Conjunctival congestion: mild (localized congestion), moderate (diffuse congestion), and severe (obvious congestion with exudation). Chemosis and conjunctival congestion should be evaluated at least one time per shift, with changes in pupils (pupil size, and light reflex) evaluated every 2-4 hours. Assessment frequency should be increased in patients with prolonged prone positioning (>12 h) or high-dose sedation. Immediate consultation with ICU physicians and ophthalmologists is required for severe signs, such as marked eyelid edema with incomplete closure, corneal exposure >1/2, or unequal pupils. |

| Continued Table 2: Construction of Eye Care Plan for Patients Receiving PPV in ICU | |
| --- | --- |
| Categories | Content of the plan |
|  |  |
|  | 4. Eye cleaning: The eyelid and eyelid margin should be gently wiped from the inner canthus to the outer canthus using cotton swabs soaked in sterile physiological saline or sterile injection water. The cleaning frequency depends on the amount of secretions, usually once every 4-6 hours. Direct contact of cotton swabs with the corneal surface should be avoided carefully to prevent mechanical damage. After eye cleaning, artificial tears or sterile Vaseline eye ointment can be applied every 6–8 hours to maintain lubrication. Eyelid abnormalities, such as trichiasis or entropion, should be assessed concurrently and managed as needed. |
|  | 5 Eye massage: For conscious and cooperative patients, active acupoint massage (Jingming, Chuzhu, Pupil Ridge, Chengxie, Sibai, Yuyao, and Sizhukong) may be performed 30–60 times per point, 2–3 times daily. For non-cooperative patients,, passive massage can be performed by nurses or family members for 10-15 minutes each time. Massage should be gentle, and direct pressure on the eyeball must be avoided to prevent increased IOP. Acupoint massage can exert functions such as eye fatigue relief, eye blood circulation improvement, as well as chemosis and congestion prevention. Following massage, the eye should be covered with a sterile transparent dressing, replaced every 4–6 hours. The dressing should be securely fixed without excessive pressure, and may be perforated to allow observation. |
|  | 6. Periocular protective pad: periocular protective pad (foam type decompression dressing) and Vaseline can be applied to the periocular skin as an emollient. |
| During PPV | 7. Position: For patients without contraindications, elevate the head of the bed to ~30° to maintain the head above heart level, thereby improving venous return and reducing facial and ocular edema. Simultaneously, adjusting head position to allow natural eyelid closure and avoid traction. Use gel headrests and foam pads to suspend the eyes and prevent orbital compression; eye protection (e.g., eye masks) may be applied if needed. Assess facial skin and ocular condition every 2 hours. Replace padding promptly if redness, swelling, or pressure injury occurs, and adjust positioning or temporarily return the patient to the supine position if necessary. |
|  | 8. Closed sputum suction: For indicated patients, a closed suction system should be connected to the endotracheal tube. Suctioning should be performed slowly and smoothly, avoiding prolonged catheter retention to reduce secretion reflux. Suction devices should be used appropriately and disposed of promptly after use. If an open system is used, precautions should be taken to prevent splashing, and any contamination around the eyes should be cleaned immediately. |

| Continued Table 2: Construction of Eye Care Plan for Patients Receiving PPV in ICU | |
| --- | --- |
| Categories | Content of the plan |
| During PPV | 9. Oral and nasal secretion cleaning: Oral care should be performed at regular intervals every day (8:00-12:00-20:00-6:00), with attention paid to keep the mouth and nose clean and moist. During oral care, the patient's head should be tilted to one side, with the use of a cotton ball/oral cleaning stick. |
|  | 10. Eye care: For patients with PPV, eye care (evaluation, and cleaning) should be performed every 4 hours. During repositioning, the eyes should be fully exposed. Turning should be conducted by at least two nurses—one stabilizing the head and neck and the other supporting the trunk—while ensuring airway patency and monitoring vital signs. The eyes should be assessed for congestion, edema, conjunctival dryness, and corneal exposure, and appropriate interventions implemented promptly. |
|  | 11. Disease observation: Vital signs should be monitored closely in patients undergoing PPV, including blood pressure, oxygenation, lactate, urine output, hemoglobin, and organ function. Abnormal findings should be reported promptly, with management adjusted accordingly. Fluid therapy and vasoactive support should be optimized, and patients should be returned to the supine position if clinically indicated. |
| After PPV | 12. Eye assessment: After PPV, eye assessment should include eyelid closure, corneal exposure, chemosis, conjunctival congestion and secretions, pupil size and light reflex, and IOP. Any abnormalities should be reported promptly, with ophthalmology consultation initiated for diagnosis and management. Nurses should assist with examinations and collaborate in implementing individualized care plans. |
|  | 13. Complication management: In cases of ocular infection, discontinue polyethylene film immediately and obtain bacterial cultures with sensitivity testing to guide targeted therapy. For closed-angle glaucoma, administer IOP-lowering agents (e.g., dexamethasone eye drops, 0.5% or 0.1% loquat eye drops, acetazolamide) with surgery if needed. Ischemic optic neuropathy requires glucocorticoids and neurotrophic therapy. Retinal occlusion should be managed with vasodilators and anticoagulants, with interventional or surgical treatment when indicated. Orbital compartment syndrome necessitates IOP-lowering therapy and possible surgery. Exposure keratitis should be treated with artificial tears and antibiotic ointment. |
|  | 14. Eye medication: It should be noted that the medication should be used at an interval of 1-5 minutes when two or more eye drops are used; The order of administration should be drip first and then ointment; and it should be applied to the fornix under the eyes, once every 2 hours. |
|  | 15. Eye cleaning, acupoint massage, and then dressing with polyethylene film should be performed for patients not experiencing any ocular complications. |
|  | 16. Quality control: Iatrogenic ocular complications should be incorporated into ICU nursing adverse event management with standardized reporting and feedback systems. Any ocular event should be managed promptly by the responsible nurse, reported within 24 hours, and followed by ophthalmology consultation. All cases should be reviewed quarterly in departmental quality meetings for root cause analysis, discussion, and continuous improvement of nursing practices. |
|  | 17. Special circumstances: During examinations (CT, MRI, etc.) or surgeries, eye protection should be taken, handover should be done with the transport and surgical personnel, eye ointment should be used for lubrication, and pads should be applied to prevent compression and pulling, with detailed recording in the medical record. |
| Training and Education | 18. Shift management: Eye condition should be included in routine shift handovers and ward rounds. Any nursing defects or complications should be promptly reported, with concurrent identification of causes and implementation of corrective measures. In addition, excellent nursing performance should be recognized and rewarded. |
